# Supplementary material for: Controllable dimensionality conversion between 1D and 2D CrCl3 magnetic nanostructures
Source: Nat Commun. 2023 Apr 28;14:2465. doi: 10.1038/s41467-023-38175-4 (PMC10147715; doi:10.1038/s41467-023-38175-4)
Supplement: Supplementary file 1 — Supplementary Information [file 41467_2023_38175_MOESM1_ESM.pdf]

Supplementary information for

Controllable dimensionality conversion between 1D and 2D $CrCl_3$ magnetic nanostructures

Shuangzan Lu[1,4#], Deping Guo[2,5#], Zhengbo Cheng[1#], Yanping Guo[1#], Cong Wang[2,5], Jinghao Deng[1], Yusong Bai[1], Cheng Tian[6], Linwei Zhou[2,5], Youguo Shi[6], Jun He[1, 3*], Wei Ji[2,5*], Chendong Zhang[1*]

[1]*School of Physics and Technology, Wuhan University, Wuhan 430072, China*

[2]*Department of Physics and Beijing Key Laboratory of Optoelectronic Functional Materials and Micro-Nano Devices, Renmin University of China, Beijing 100872, China*

[3]*Wuhan Institute of Quantum Technology, Wuhan 430206, China*

[4]*Hubei Jiufengshan Laboratory, Wuhan 430074, China*

[5]*Key Laboratory of Quantum State Construction and Manipulation (Ministry of Education), Renmin University of China, Beijing, 100872, China*

[6]*Beijing National Laboratory for Condensed Matter Physics and Institute of Physics, Chinese Academy of Sciences, Beijing 100190, China*

[#]*These authors contribute equally to this work.*

**Correspondence and requests for materials should be addressed to: He-jun@whu.edu.cn (J. H.), wji@ruc.edu.cn (W. J.), cdzhang@whu.edu.cn (C.D. Z)*

Supplementary Figures S1–S13

Supplementary Tables S1–S3

Supplementary Figures

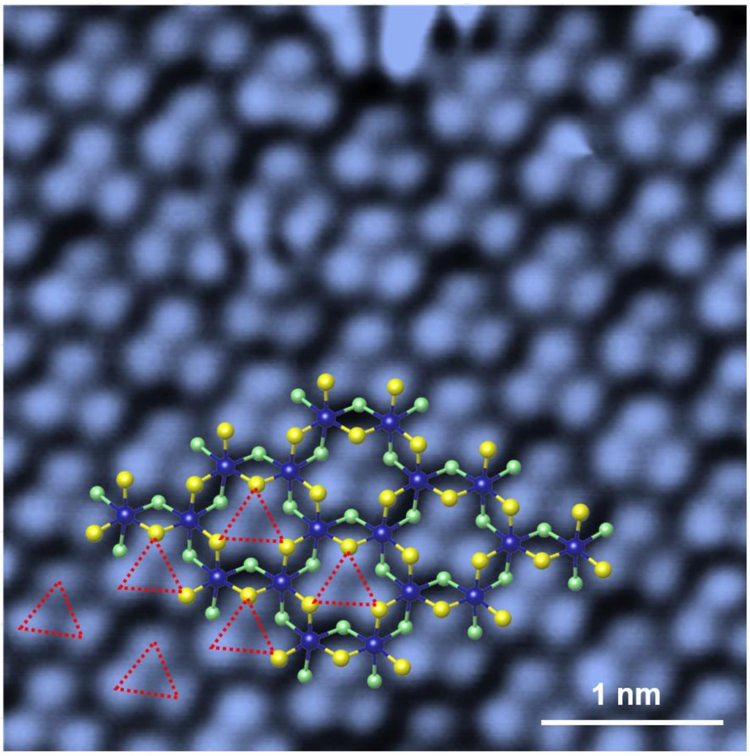

Figure S1 | Atomically resolved image of the small $CrCl_3$ flake on $NbSe_2$. The image was taken on the small 2D flake that joints the wires (Fig. 1b). The dashed red triangles indicate the Cl trimers imaged by STM. It confirms that these small 2D flakes obtained on $NbSe_2$ are in the 2D phase. Scanning parameters: 0.1 V, 50 pA.

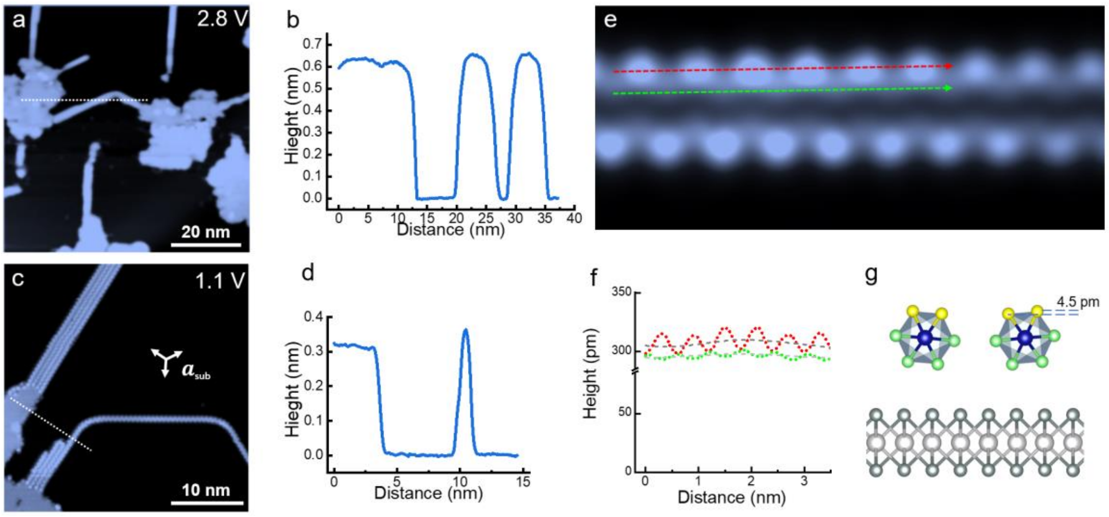

Figure S2 | Additional information for the 1D and 2D $CrCl_3$ phases on $NbSe_2$. a, STM image obtained at the bias voltage of 2.8 V. **b**, The line profile along the dashed line in **a** shows that the heights of the 1D-wire and 2D-flake are about 6.2 Å. **c**, STM image obtained at the bias voltage of 1.1 V. The line profile in **d** (taken along the dashed line in **c**) illustrates that at 1.1 V, the apparent height of 1D wire is slightly higher than that of the 2D flake. **e**, In the parallelly stacked bi-wires, the two rows of top-most Cl atoms in each wire are not of the equivalent height. **f**, The line profiles [paths are labeled in **e**] show an average height difference of ~ 9 pm between the two Cl atoms rows. The gray dashed lines represent the center levels of the green and red height profiles. **g**, The calculated height difference of the two rows of Cl atoms is marked as 4.5 pm, which is consistent well with the experimental value.

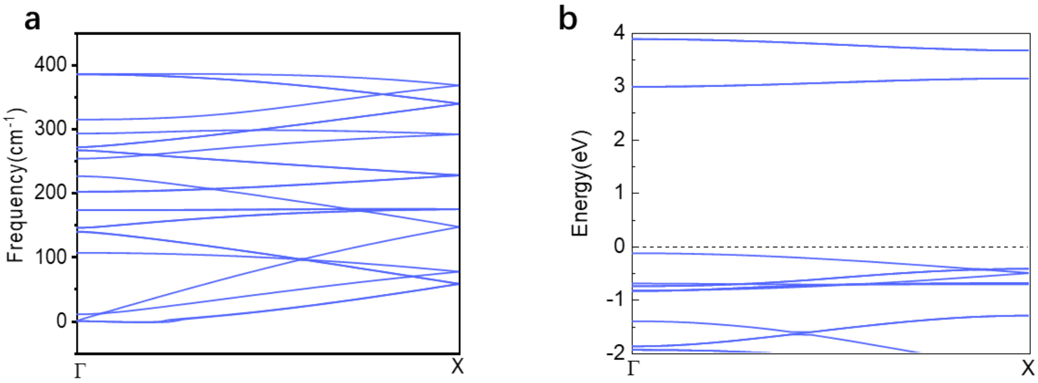

Figure S3 | The phonon spectrum and band structure of freestanding 1D $CrCl_3$ single wire. **a**, The phonon spectrum of freestanding 1D $CrCl_3$ single wire. **b**, Calculated electronic band structure of single wire (freestanding, infinite length) in Néel AFM ground state.

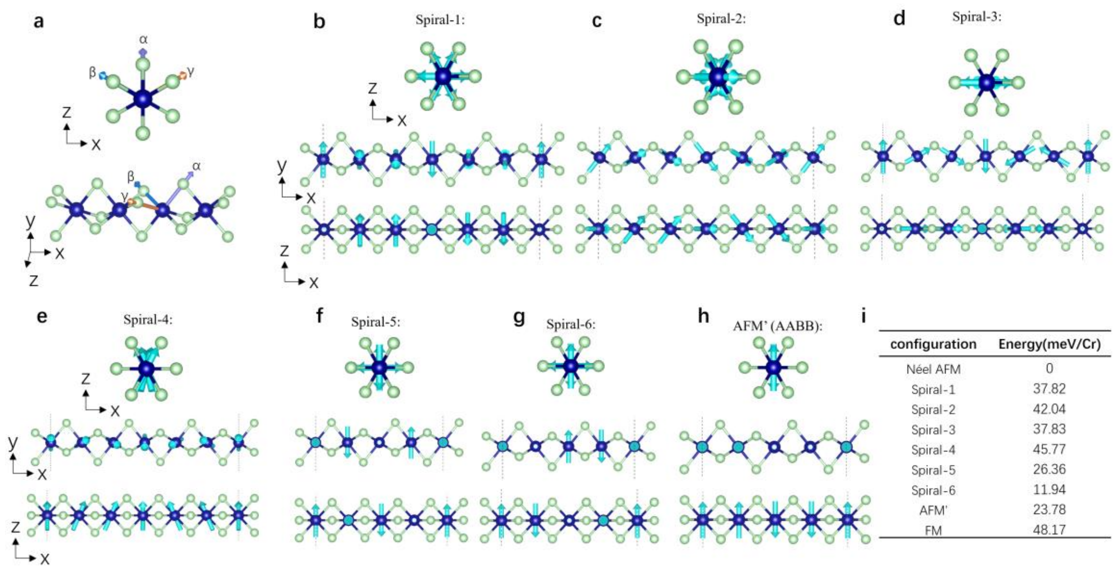

configuration	Energy(meV/Cr)
Néel AFM	0
Spiral-1	37.82
Spiral-2	42.04
Spiral-3	37.83
Spiral-4	45.77
Spiral-5	26.36
Spiral-6	11.94
AFM'	23.78
FM	48.17

Figure S4 | Examination of the preference in forming spiral magnetism. a, Illustration of the Kitaev local basis $\{\alpha, \beta, \gamma\}$. **b-h**, Schematics of seven magnetic configurations of 1D $CrCl_3$ single wire including spiral-1, spiral-2, spiral-3, spiral-4, spiral-5, spiral-6 and AFM'(AABB). **i**, Total energy comparison among various magnetic configurations respect to the Néel AFM (ABAB) state. It supports that the SWs energetically favor the collinear Néel AFM state.

Non-collinear spin-exchange parameters were derived to examine whether a spiral magnetism is preferred to form. An inversion center exists in periodic $CrCl_3$ SWs, which eliminates the Dzyaloshinski–Moriya (DM) interaction in the SWs.

To derive Kitaev parameters K and Γ, we used a Hamiltonian as $H=-\frac{1}{2}\sum_{ij}\boldsymbol{S}_{\mathbf{i}}\cdot J_{ij}\cdot\boldsymbol{S}_{\mathbf{j}}+\sum_{ij}\boldsymbol{S}_{\mathbf{i}}\cdot A_{ii}\cdot\boldsymbol{S}_{\mathbf{j}}+H_0$, where i and j represent two nearest neighboring sites of Cr atoms, J_{ij} and A_{ii} are exchange and single ion anisotropy parameters, respectively. H_0 is the energy term except for the exchange interactions. By considering many magnetic configurations, we derived the general matrix in the $\{x, y, z\}$ basis set as

$$\boldsymbol{J}=\begin{pmatrix}-10.67 & 0 & -0.54\\ 0 & -9.61 & -0.01\\ -0.54 & -0.01 & -10.66\end{pmatrix}$$

The nearest isotropic parameter J_1 was defined as $(J_{xx}+J_{yy}+J_{zz})/3=-10.31$ meV/Cr. The J matrix is next diagonalized in local basis $\{\alpha, \beta, \gamma\}$, as illustrated in panel **a**, which are along the Cr-Cl bonding direction. Under this basis set, the J matrix reads

$$\boldsymbol{J} = \begin{pmatrix} -10.05 & -0.13 & 0.17 \\ -0.13 & -9.96 & -0.58 \\ 0.17 & -0.58 & -10.92 \end{pmatrix},$$

Thus, K is derivable from $K = J_\gamma - (J_\alpha + J_\beta)/2 = -0.92$ meV/Cr [1, 2], which is much smaller than the isotropic Heinsberg parameter J_1 ($K/J_1 \approx 0.09$). The non-diagonal term Γ are even smaller than K. The small K/J and Γ/J ratios indicate the Kitaev interactions are more prefer collinear magnetism.

In addition, Heisenberg J_1-J_2 model was considered in a Hamiltonian $H = -\frac{1}{2}J_1\sum_{ij}\boldsymbol{S}_i \cdot \boldsymbol{S}_j - \frac{1}{2}J_2\sum_{<ij>}\boldsymbol{S}_i \cdot \boldsymbol{S}_j + H_0$ where ij and $<ij>$ represent the nearest and the next nearest neighboring sites of Cr atoms, respectively. By fitting different magnetic configurations, we derived $J_1 = -10.72$ meV/Cr, $J_2 = 0.83$ meV/Cr, which lead to a J_1/J_2 ratio over 11, explicitly favoring the collinear antiferromagnetic coupling.

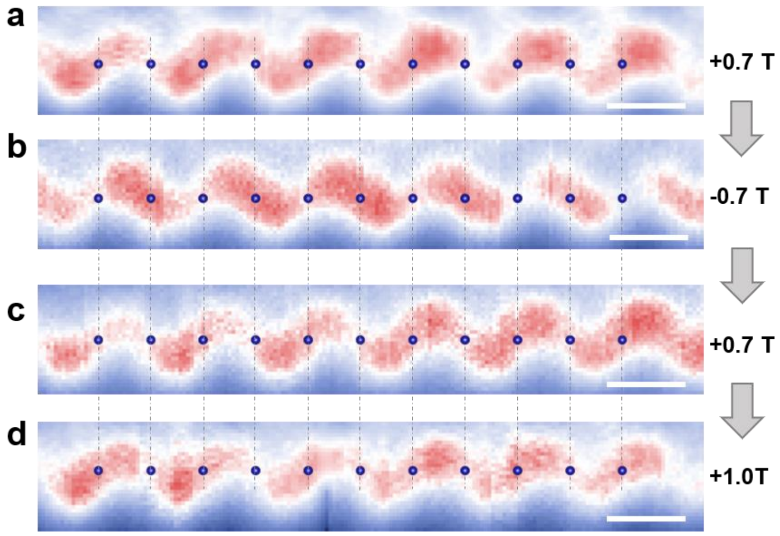

Figure S5 | Cross-check of the contrast reversal imaged by SP-STM. The external fields used to magnetize the tip are labeled as shown. The arrows represent the sequence of data acquisitions. **a** and **b** are the same ones as Fig. 2h. After that, the tip magnetization was flipped back to the spin-up state by applying positive magnetic fields (**c**, +0.7 T; and **d**, +1.0 T). **c** and **d** well reproduced the topographic feature in **a**. Scale bars are 0.4 nm.

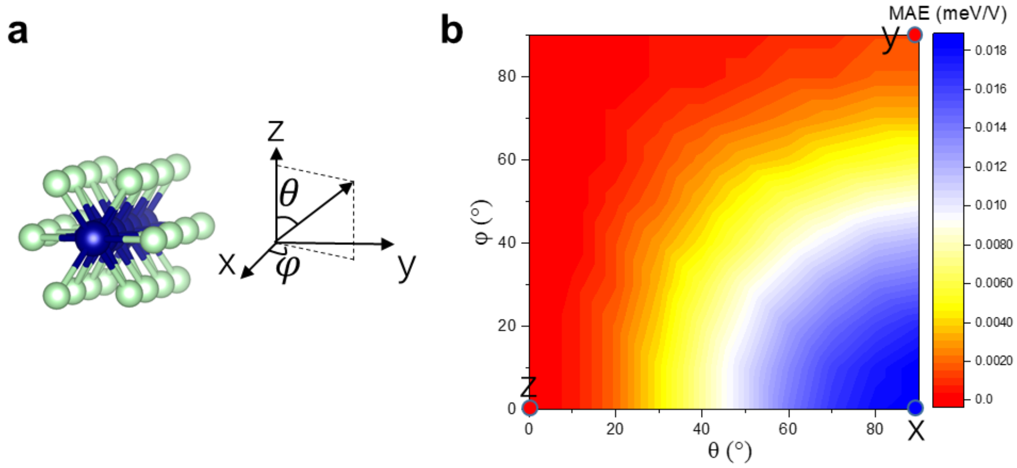

Figure S6 | Magnetic anisotropy energy of the freestanding $CrCl_3$ single wire. a, As discussed in Fig. S4, the single wire prefers the AFM(ABAB) state. The direction of the magnetic moments (spin-up) was defined by the angles θ and φ (hence the spin-down direction refers to $\pi - \theta$ and $\pi + \varphi$). The coordinate system was defined as shown in Fig. 2g, where the wire axis is along *x*. **b**, Color-coded mapping of the magnetic anisotropy energy (MAE) in the single wire with an infinite length. The color scale bar is displayed as shown. The ranges of θ and φ are 0-90º. When the magnetic moments are perpendicular to the single wire (*i.e.*, within the *yz* plane), the system has the lowest energy. And highest energy occurs when the magnetic moments are along the wire axis (*x*). Thus, the easy axis of the single wire locates within the *yz* plane with a MAE of about 0.02 meV/Cr. Upon adsorption on the $NbSe_2$ surface, the easy-plane degrades, most likely, into an easy axis along z, which is 0.005 meV/Cr favored than the *y*-direction. Noted the magnitude of MAE in the $CrCl_3$ wire is relatively small (particularly between the *z*- and *y*- direction), while the AFM coupling is strong (see discussions in Fig. S4). In our SP-STM measurements, the magnitude of the spin-contrast taken by a *z*-polarized tip can show variations in a long distance, which is consistent with the small MAE between *z and y* directions arising from the adsorption on $NbSe_2$.

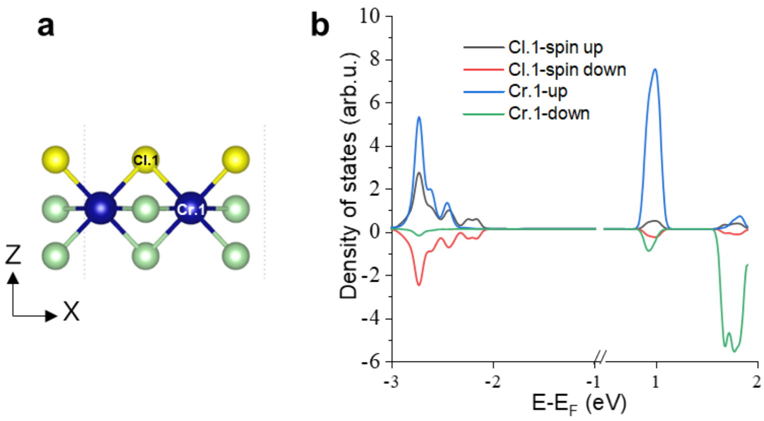

Figure S7 | Spin and atom resolved theoretical DOS of a $CrCl_3$ SW on the $NbSe_2$ substrate. a, An atomistic model of the $CrCl_3$ wire with labels of the Cl.1 and Cr.1 atoms. Cl.1 is a top layer atom. The $NbSe_2$ substrate is not shown here. **b**, Plots of spin-resolved DOS projected on the Cl.1 and Cr.1 atoms. The two spin components on Cl.1 show a rather slight difference. However, the DOS on Cr.1 are significantly polarized, with a maximum polarization occurring around the conduction band (CB) edge (*i.e.*, +1.0 V in calculations). This result guided us to choose the sample bias used in the SP-STM studies. The experimental CB edge locates around +1.3 V (Fig. 2f), and we used +1.5 V in Fig. 2h for tip safety.

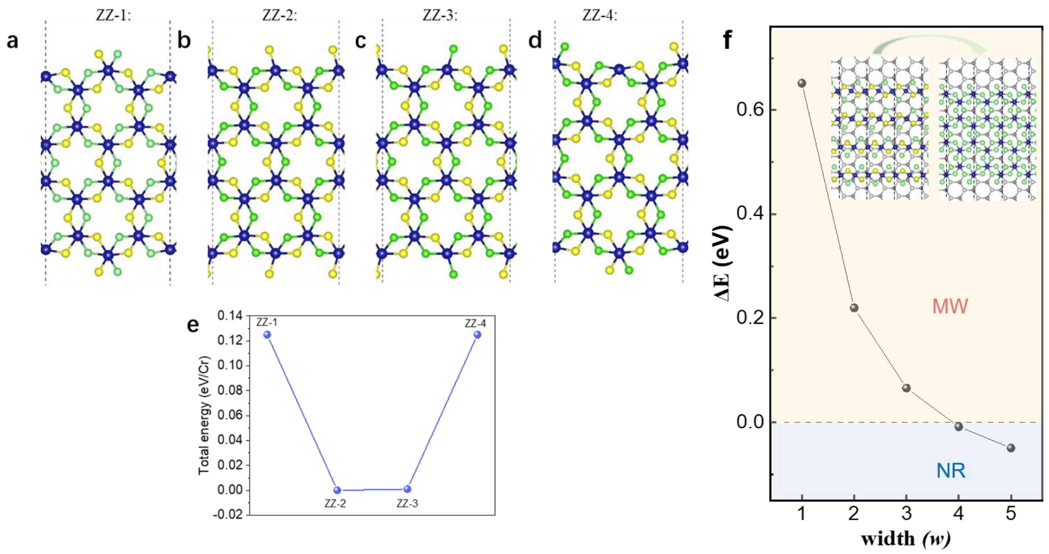

Figure S8 | Calculations of the MW-NR transformation observed in Fig. 3g. To reproduce the experimental observations in Fig. 3g, we need to build a model that satisfies the requirement of conservation of atoms during the transformation. This requirement is grounded in the following observations. First, inside the ribbon, the lattice is identical to that of the 2D $CrCl_3$ film. Throughout our experiments, we observed no additional clusters/atoms in the adjacent area after the tip-induced transformation, even at an ultra-low temperature (0.4 K). This indicates that the atomic number was well conserved. In other words, half of the edge Cl atoms need to be removed in the model of NRs that are *in situ* converted from MWs. Second, the two edges of the ribbon are identical in morphology; neither of them shows a signature of reconstruction. Therefore, four possible edge structures can be proposed, with their schematic atomic models (for w = 4) displayed in **a–d**. The green and yellow atoms represent the Cl atoms at the bottom and top layers, respectively. The blue atoms represent the Cr atoms. **e** shows a plot of the total energies for all four models. ZZ-2 and ZZ-3 models with Cl atoms removed for each edge unit are significantly more stable than ZZ-1 and ZZ-4 (with 2×1 edges). **f** shows the difference in the total energies of the two phases ($\Delta E = E_{NR} - E_{MW}$) as a function of the width w. The model we adopted here (inset) is ZZ-2, which has a slightly lower total energy than ZZ-1. The experimental estimation of the threshold width was well reproduced by our calculations, where ΔE crossed zero between w = 3 and 4.

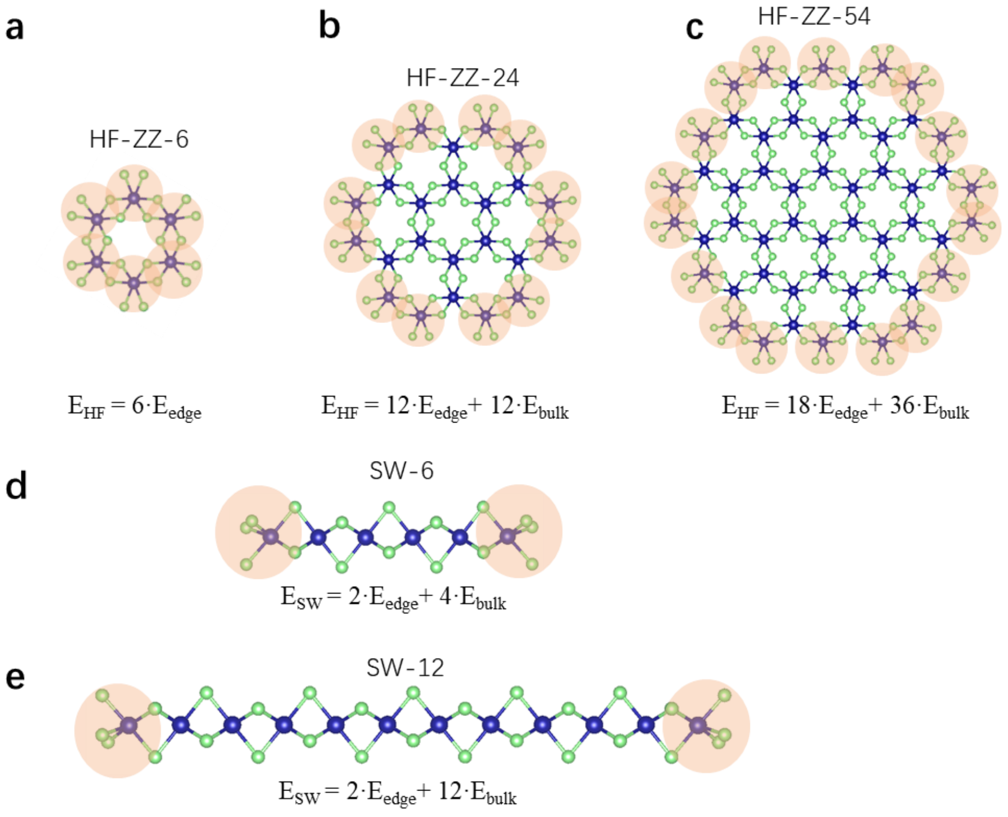

Figure S9 | Atomic structures of the hexagonal flakes and SWs. Figure 4c in the main text shows that the HFs with zigzag edges (*i.e.*, $n_{Cr} = 6$, 24, and 54) are more stable than those arm-chair ones ($n_{Cr} = 12$ and 36). **a–c**, Top views of the more stable hexagonal flakes (HF-ZZ) in the 2D phase with n_{Cr} = 6, 24 and 54, respectively. **d–e**, Top views of the single wires (SW) with n_{Cr} = 6 and 12, respectively. The edge units are marked by orange shadows. The atomic structures show that the ratios between the end (edge) and bulk units are size-dependent and are different in SW and HF-ZZ. The SW phase only has two end units at any length, and the bulk unit is dominant even at a short length, *e.g.*, wire Cr_6Cl_{21} contains 2 $CrCl_{4.5}$ end and 4 $CrCl_3$ bulk units. In the HF-ZZ phase, however, the number of edge units is comparable to or even larger than that of the bulk units in small-size HFs, *e.g.*, HF-ZZ Cr_6Cl_{24} contains 6 $CrCl_4$ edge units and HF-ZZ $Cr_{24}Cl_{84}$ has 12 $CrCl_4$ edge and 12 $CrCl_3$ bulk units.

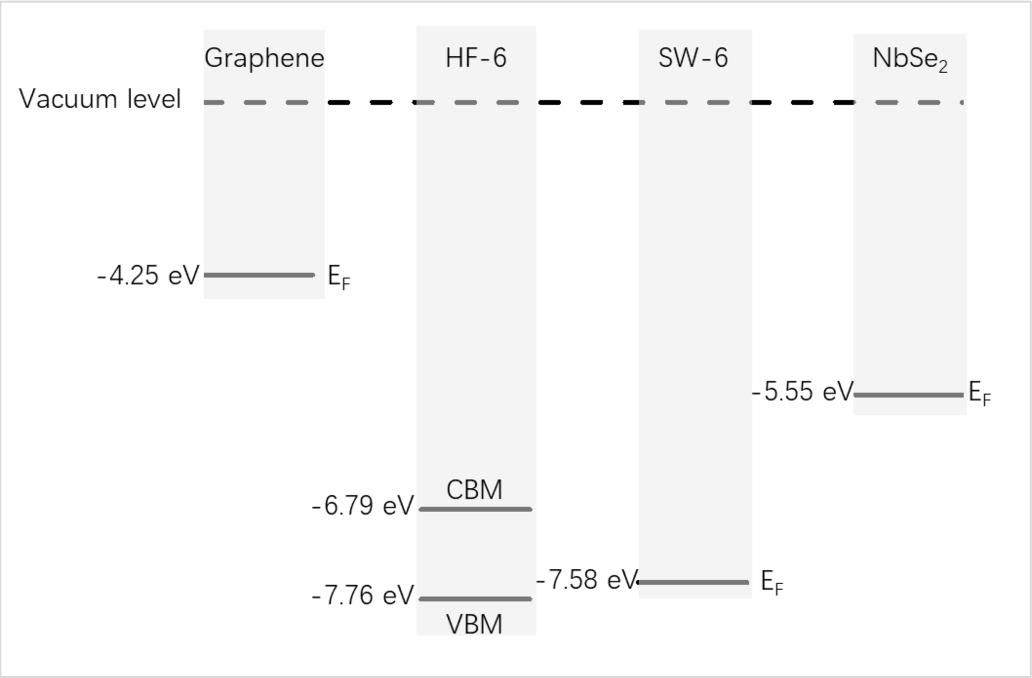

Figure S10 | Critical energy levels of the HF-6, SW-6, and the two types of substrates. All energy values correspond to the freestanding situations and are relative to the vacuum level. E_F is the Fermi energy level, and VBM (CBM) corresponds to the valence band maximum (minimum conduction band). Strictly speaking, the freestanding SW-6 has a non-zero density of states at the E_F. Hence, only the Fermi energy level is given here for the SW-6. The critical energy levels of SW-6/HF-6 are significantly lower than the E_F of the graphene and $NbSe_2$. Moreover, the graphene (4.25 eV) has a smaller work function than the $NbSe_2$ (5.55 eV), yielding more electrons transferring from graphene into the on-top nanostructures.

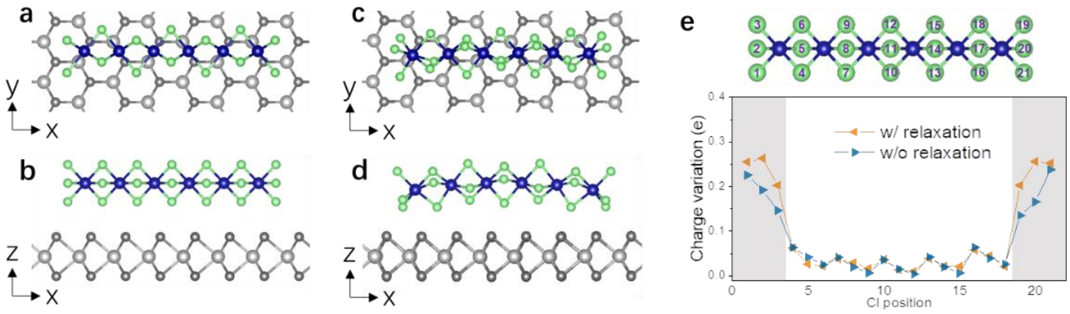

Figure S11 | Atom-specified charge transfer between SW-6 and $NbSe_2$ without/with structural relaxation. a-b, Top and side view of SW-6 on $NbSe_2$ without structural relaxation. **c-d**, Top and side view of SW-6 on $NbSe_2$ with structural relaxation. **e**, Charge variation in corresponding position of SW-6 on $NbSe_2$ without and with structural relaxation. Unsaturated Cl atoms (Cl dangling bonds) at the ends or edges primarily lead to substantial charge transfer from the substrate to SW or HF-ZZ predominately occurring at the ends or edges. Each end (edge) unit contains a $CrCl_{4.5}$ ($CrCl_4$) group, which needs one or one and a half electron to fully saturate, given the valence of 3+ (1-) for Cr (Cl). Thus, the ends and edges are strong electron acceptors. However, their bulk counterparts both consist of $CrCl_3$ groups, showing comparable chemical potentials with the $NbSe_2$ and graphene substrates. In light of this, structural relaxation plays a minor and passive role in the charge transfer.

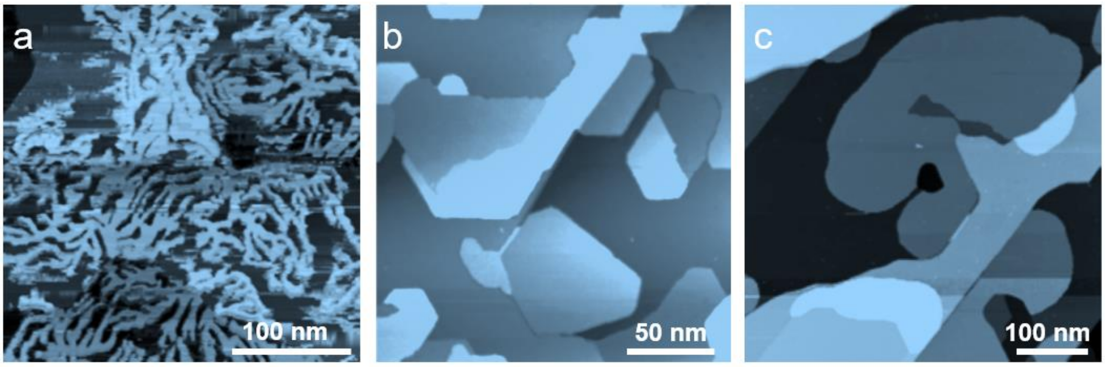

Figure S12 | Substrate temperature (T_{sub}) dependent morphologies of $CrCl_3$ grown on epitaxial bilayer graphene. **a–c**, T_{sub} = room temperature, 500 K and 540 K. Here, T_{sub} refers to the substrate temperature holding during the deposition of $CrCl_3$. The fractal-like patterns form at T_{sub} = room temperature. As raising of the T_{sub}, it tends to form compact islands with straight edges along the high symmetric directions. Once the T_{sub} is over 510 K, the straight edges start to merge, and round-shape islands appear. The observed morphological transition indicates the growth of $CrCl_3$ on BLG follows the classical diffusion-limited aggregation (DLA) model. Noted that, at all above growth conditions, only the 2D phase atomic structure was observed.

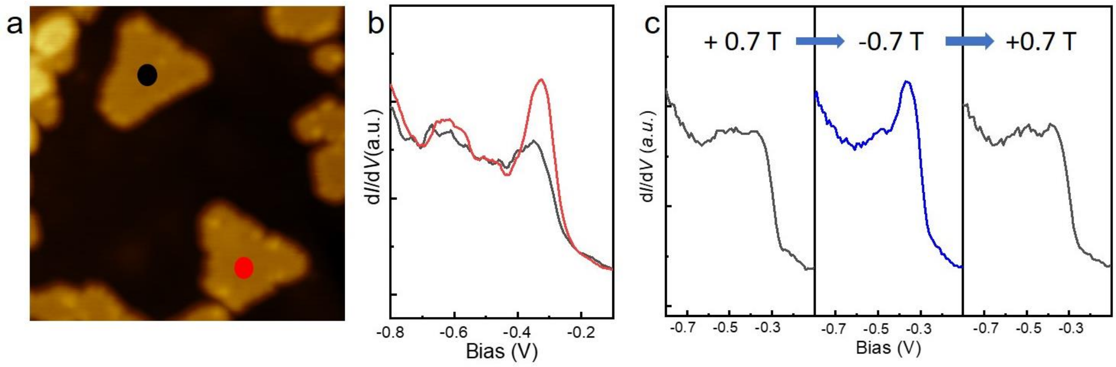

Figure S13 | Calibration of spin-polarized Ni tip on Co/Cu(111). a, A STM image showing the topography of Co islands on Cu(111). Scanning parameters: 1 V, 10 pA, 36 × 36 nm. **b,** d*I*/d*V* spectra obtained on two Co islands using the pre-magnetized (by B = + 0.7 T) Ni tip. The black and red curves are taken at the black and red spots in **a**, respectively. **c,** A series of d*I*/d*V* spectra (on the black spot) with the positive and negative external fields alternatively applied. All other parameters for these spectroscopic measurements are the same. Note that the magnetic moments of Co islands will not flip unit |B| > 1T [3]. Therefore, we confirmed that the magnetization of this Ni tip can be repeatedly flipped by perpendicular magnetic fields. The flip of the spatial contrast illustrated in Fig. 2h shows the same reversible behavior, supporting the similar tip-magnetization process occurred on the $CrCl_3/NbSe_2$ surface.

Supplementary Tables

Band gap of $CrCl_3$ (eV)	U=3.9, J=1.1	
	SW	2D
Infinite w/o $NbSe_2$	3.12	3.17
Infinite w/ NbSe2	2.81	3.08
Finite (n_{cr}=6) w/o $NbSe_2$	0.75*	0.97
Finite (n_{cr}=6) w/ $NbSe_2$	1.25	1.52

Table S1 | Theoretical energy gap between the edges of valence and conduction states, respectively, of $CrCl_3$ in various forms. Introduction of the $NbSe_2$ substrate leads to metallicity of the adsorbed systems, in which we count the energy differences between the VB and CB band edges of $CrCl_3$ solely. Those energy gaps were derived from the projected density of states onto the on-top $CrCl_3$ nanostructures. With $U = 3.9$ eV and $J = 1.1$ eV, the gap of infinite 2D monolayer $CrCl_3$ (w/ $NbSe_2$) is 3.08 eV, which is larger than that of the corresponding single wire (2.81 eV). Because of the partially occupied edge states, the VB of free-standing SW-6 has a band tails across E_F, in which the gap value is derived exactly following the edge-to-edge definition and marked with a star symbol.

(eV)		FS	On $NbSe_2$	On Gr
SW<br>$CrCl_{4.5}$ (end)<br>$CrCl_3$ (bulk)	H^{SW}_{edge} (eV/Cr)	-8.43	-9.89	-10.37
	H^{SW}_{bulk} (eV/Cr)	-9.90	-10.23	-10.13
HF-ZZ<br>$CrCl_4$ (edge)<br>$CrCl_3$ (bulk)	$E^{HF\text{-}ZZ}_{edge}$ (eV/Cr)	-9.27	-10.13	-10.42
	$E^{HF\text{-}ZZ}_{bulk}$ (eV/Cr)	-10.22	-10.56	-10.41
$\boldsymbol{E^{HF\text{-}ZZ}_{edge} - E^{SW}_{bulk}}$ **(eV/Cr)**		**0.63**	**0.10**	**-0.29**

Table S2 | Decomposed energies onto the edge and bulk $CrCl_x$ units of SW and HF-ZZ. For freestanding cases, see Fig. S9 for their atomic structures, the total formation energy of each $CrCl_3$ structure could be represented by the summation of the energies of each bulk and edge units. For example, for SW-6, its $E_{total\_SW\text{-}6} = 2 \cdot E_{edge} + 4 \cdot E_{bulk}$; and for SW-12, one gets $E_{total\_SW\text{-}12} = 2 \cdot E_{edge} + 10 \cdot E_{bulk}$. Energies E_{edge} and E_{bulk} of the SW from are derivable by solving this equation set. Similar calculations were performed for the freestanding HF, where $E_{total\_HF\text{-}6} = 6 \cdot E_{edge}$, and $E_{total\_HF\text{-}24} = 12 \cdot E_{edge} + 12 \cdot E_{bulk}$ for HF-6 and HF-24, respectively. In the presence of substrates, the energy of each type of units includes the total energy of the unit and its adsorption energy on the substrate, which was done by solving the equation set after subtracting the substrate energy from the total energy of the adsorbed system.

The SW phase only has two end units at any length and the bulk unit is dominant even at a short length. In the HF-ZZ phase, however, the number of edge units is comparable or even larger than that of the bulk units in small-size HFs. We listed the values of ($E^{SW}_{bulk} - E^{HF\text{-}ZZ}_{edge}$). It clearly shows that the SW could be more preferred (negative value) in the free-standing form or on $NbSe_2$ under a certain range of Cl chemical potential, but is inaccessible on graphene.

$CrCl_{3+X}$	Finite (eV/Cr)		Infinite (eV/Cr)
	end/edge	bulk	
1D chain	-1.46	-0.33	-0.30
2D phase	-0.86	-0.34	-0.31

Table S3 | Adsorption energy of 1D wire and 2D phase on $NbSe_2$ in infinite and finite cases. The adsorption energy of a finite $CrCl_3$ chain (flake) is decomposed into a bulk and an end (edge) term. Table shows that bulk term values are highly comparable ranging from -0.30 to -0.34 eV/Cr for finite and infinite forms. However, the adsorption energy of the end and edge units are much larger, saying -1.46 and -0.86 eV/Cr for the 1D and 2D forms. These significant adsorption energies, together with the DFT revealed large charge transfer, support the mechanism that the substrate strongly interacts with the ends (edges) of $CrCl_3$ chain (flake) to screen their Cl dangling bonds.

Supplementary References

1. Xu, C. et al. Topological spin texture in Janus monolayers of the chromium trihalides $Cr(I, X)_3$. *Phys. Rev. B* **101**, 060404 (2020).

2. Xu, C., Feng, J., Xiang, H. & Bellaiche, L. Interplay between Kitaev interaction and single ion anisotropy in ferromagnetic CrI3 and CrGeTe3 monolayers. *npj Comput. Mater.* **4**, 57, (2018).

3. Rodary, G., Wedekind, S., Sander, D. & Kirschner, J. Magnetic Hysteresis Loop of Single Co Nano-islands. *Jpn. J. Appl. Phys.* **47,** 9013 (2008).
